# Body Composition and Demographic Features Do Not Affect the Diagnostic Accuracy of Shear Wave Elastography

**DOI:** 10.3390/bioengineering10080904

**Published:** 2023-07-30

**Authors:** Umut Varol, Juan Antonio Valera-Calero, César Fernández-de-las-Peñas, Jorge Buffet-García, Gustavo Plaza-Manzano, Marcos José Navarro-Santana

**Affiliations:** 1 Escuela Internacional de Doctorado, Universidad Rey Juan Carlos, 28922 Alcorcón, Spain; au.varol.2022@alumnos.urjc.es; 2 Department of Radiology, Rehabilitation and Physiotherapy, Faculty of Nursery, Physiotherapy and Podiatry, Complutense University of Madrid, 28040 Madrid, Spain; gusplaza@ucm.es (G.P.-M.); marconav@ucm.es (M.J.N.-S.); 3 Grupo InPhysio, Instituto de Investigación Sanitaria del Hospital Clínico San Carlos (IdISSC), 28040 Madrid, Spain; 4 Cátedra Institucional en Docencia, Clínica e Investigación en Fisioterapia: Terapia Manual, Punción Seca y Ejercicio Terapéutico, Universidad Rey Juan Carlos, 28922 Alcorcón, Spain; cesar.fernandez@urjc.es; 5 Department of Physical Therapy, Occupational Therapy, Rehabilitation and Physical Medicine, Universidad Rey Juan Carlos, 28922 Alcorcón, Spain; 6 Faculty of Health Sciences, Universidad Francisco de Vitoria, 28223 Pozuelo de Alarcón, Spain; j.buffet.prof@ufv.es

**Keywords:** anterior scalene, diagnostic accuracy study, reliability, shear wave elastography, ultrasonography

## Abstract

Shear-wave elastography (SWE) is an imaging method that can be used to estimate shear wave speed and the Young’s modulus based on the measured shear wave speed under certain conditions. Up to date, no research has analyzed whether body composition factors contribute to ultrasound attenuation, refraction, reflection, and, consequently, SWE measurement errors. Therefore, this study aimed to analyze the association between demographic and body composition features with SWE errors for assessing the anterior scalene stiffness (which is a key structure in patients with neck pain and nerve compressive syndromes). Demographic (sex, age, height, weight, and body mass index), body composition (water volume, fat mass, and lean mass), and anterior scalene muscle stiffness (Young’s modulus and shear wave speed) data were collected from a sample of asymptomatic subjects. After calculating the absolute SWE differences between trials and the reliability estimates, a correlation matrix was generated to quantify the association among all the variables. A total of 34 asymptomatic subjects (24 males) were included in the analyses. Test–retest reliability was excellent for assessing the Young’s modulus and shear wave velocity (ICC = 0.912 and 0.923, respectively). No significant associations were found between age, height, weight, body mass index, body fat, lean mass, or water volume with SWE errors (*p* > 0.05). However, the Young’s modulus error was associated with the stiffness properties (*p* < 0.01), whereas shear wave speed was associated with none of them (all, *p* > 0.05). A detailed procedure can reliably assess the AS muscle stiffness. None of the sociodemographic or body composition features assessed were correlated with SWE errors. However, baseline stiffness seems to be associated with Young’s modulus error.

## 1. Introduction

There is a growing body of scientific research focused on the evaluation of muscle stiffness due to its potential as a discriminative factor between asymptomatic individuals and clinical populations [1,2,3,4]. This parameter has garnered particular attention in relation to neurological populations, as neurological conditions and neuromuscular dysfunctions often manifest with notable alterations in muscle mechanics and morphology [1]. Moreover, muscle stiffness assessment is also relevant in various musculoskeletal conditions characterized by muscle tissue damage, such as calf injuries [2]. 

In such cases, fibrotic processes occur, which involve the excessive formation of connective tissue between skeletal muscle fibers, leading to the development of muscle scars [3]. By evaluating muscle stiffness, clinicians and researchers can gain valuable insights into the extent of fibrotic tissue formation and the subsequent impact on muscle function and recovery. However, muscle stiffness assessment is not limited to conditions involving muscle damage. It is also employed in the evaluation of disorders such as myofascial pain syndrome [4], where muscle stiffness can provide important diagnostic information and help guide treatment approaches. Additionally, muscle stiffness assessment plays a role in studying age-related muscle loss and weakness, known as sarcopenia [5]. By quantifying muscle stiffness in individuals with sarcopenia, researchers can better understand the mechanical changes occurring in the muscle tissue and potentially identify interventions to mitigate its progression.

Ultrasound elastography is used for acquiring tissue elasticity information in this context. It serves as an adjunct to B-mode ultrasound imaging (US) in order to detect early stages of disease when no morphological abnormalities can be depicted with grey-scale [6]. However, different elastography methods for assessing musculoskeletal structures are described in the literature [7]. Strain elastography was the first method developed and uses the tissues’ deformation induced by an internal (e.g., a vessel pulse) or external force (e.g., using the transducer) to calculate the tissues’ longitudinal displacement before and after compression. This method provides relative information to calculate strain ratios (index of relative elasticity between two areas) and creates an elasticity color map (using a color scale to visualize a translucent colored elastogram), using the tissue suffering the highest (softest region) and the lowest deformation (stiffest region) as references [8]. 

On the other hand, shear wave elastography utilizes mechanical shear waves produced by the compressive acoustic waves used for acquiring the B-mode images, as shear wave propagation velocity depends on tissue stiffness. In contrast with strain elastography, this is an absolute unit expressed in m/s, which can be algebraically transformed to calculate the Young’s modulus in kPa to calculate the absolute pressure needed to deform a tissue [9]. 

Due to the differences in physics principles, SWE overcomes several limitations of strain elastography. Strain elastography is highly subjective since the external and internal forces are difficult to standardize, the magnitude of the stiffness difference between two areas is unknown, and longitudinal assessments are limited [10]. However, it should be noted that both methods are based on ultrasound waves and associated artifacts such as attenuation, shadowing, anisotropy, and the location of the region of interest may equally influence the results reproducibility in both modalities [11]. The rationale for conducting this study is that limited evidence has analyzed if non-operator-dependent factors such as patients’ demographics and body composition characteristics are associated with increased SWE errors, despite the large number of studies assessing the test–retest reliability of SWE in calculating the stiffness properties of musculoskeletal structures [12,13,14,15,16,17,18]. 

A previous study [4] analyzing the association between Young’s modulus and shear wave speed at three different locations of the upper trapezius muscle (active myofascial trigger points—a hypersensitive tender spot located in a muscle taut band, which, under manual palpation stimulation, reproduces partially or totally familiar symptoms experienced by the patients—or latent myofascial trigger points—a hypersensitive tender spot located in a muscle taut band painful to manual palpation, but not recognized as familiar- and control locations). However, the Young’s modulus and shear wave speed differences among active myofascial trigger points, latent trigger points, and control locations within the muscle revealed that clinical populations with chronic, non-specific neck pain show greater general stiffnesses compared to asymptomatic controls [4].

As the anterior scalene (AS) muscle is a key location with high clinical relevance, as most of the thoracic outlet syndromes result from neurovascular compression in the interscalene space (delimited by the first rib, the anterior and medium scalene muscles) [19,20], it is essential to identify the factors associated with SWE errors in this area. This identification will help confirm which factors contribute to lower diagnostic accuracy and consider them in further studies that analyze differences between clinical and asymptomatic populations or examine the association between muscle fibrosis or the presence of myofascial trigger points with clinical severity indicators. 

Therefore, the aims of this study were to calculate the test–retest reliability of a novel procedure for estimating the AS muscle stiffness using SWE and to analyze the association between the stiffness metrics error (i.e., shear wave speed error and Young’s modulus error) with participants’ characteristics, including sociodemographic (i.e., sex, age, height, weight, and body mass index), body composition (i.e., lean mass, fat mass and water volume), and stiffness features (i.e., shear wave speed and Young’s modulus).

## 2. Materials and Methods

### 2.1. Study Design

Between October 2022 and March 2023, a diagnostic accuracy designed cross-sectional observational study was conducted for assessing the test–retest reliability of a novel procedure targeting the AS muscle stiffness assessment, and the association between SWE errors with participants’ characteristics was conducted at a private university located in Ávila (Spain). In order to enhance the quality of this research, the writing adhered to the Reporting Reliability and Agreement Studies (GRRAS) guidelines [21] and the Enhancing the Quality and Transparency Of Health Research (EQUATOR) guidelines [22]. The entire protocol was supervised and approved by the Ethics Committee of Rey Juan Carlos University prior to data collection.

### 2.2. Participants

After posting local announcements around the campus, a sample consisting of asymptomatic volunteers was recruited through convenience sampling. Volunteers between the ages of 18 and 65 who had not experienced any neck pain symptoms in the past year were considered eligible for participation. No strict limits for weight, height, body mass index, or body composition metrics were imposed to improve the results’ generalizability. Those participants reporting previous history of whiplash, taking medication that may alter the muscle tone (such as muscle relaxants), underwent any surgical procedure, having any neuropathic condition (such as radiculopathy, thoracic outlet syndrome, or myelopathy), or demonstrating severe degenerative radiologic findings were excluded from the study. All participants meeting the eligibility requirements were asked to read and sign an informed written consent prior to their participation in the data collection.

### 2.3. Sample Size Calculation

The minimum sample size for this study was estimated following the guidelines presented by Harris for correlation-based designs (*n* = 50 + number of variables), as this calculation has demonstrated acceptable validity and power in detecting associations and conducting factor analyses [23,24]. As the number of independent variables included in the correlation matrix was set at 11, this research required a minimum of 66 data points to achieve acceptable statistical power. 

### 2.4. Assessments

#### 2.4.1. Demographic and Body Composition Features

A standardized self-reported form was filled out by all participants, including information about their age, sex, and height. For assessing the remaining demographic and body composition data (e.g., water volume, weight, body mass index, body fat, and lean mass), an InBody 270 bioimpedance device (Biospace, Urbandale, IA, USA) was used. This device was selected since the literature described an almost perfect correlation between the bioimpedance scores with Gold Standard methods such as dual-energy X-ray absorptiometry and excellent reliability [25]. A laboratory technician participated as an independent investigator carrying out all the measurements, weighing all participants in light clothing between 9:00 am and 11:00 am, as described by the authors in their validity study [25].

#### 2.4.2. Anterior Scalene Muscle Stiffness

A single examiner with +10 years of experience in the use of musculoskeletal US and clinical practice conducted all US measurements using a Logiq E9 device with a 6–15 MHz ML-6-15-D linear transducer (General Electric Healthcare, Milwaukee, WI, USA). The console settings were standardized for all the imaging acquisitions (Frequency = 12 MHz, Gain = 65 dB and Depth = 4.5 cm). This examiner performed two measurements (each one spaced by 2 h) in order to obtain reliability estimates and analyze the correlation of test–retest error with body composition and sociodemographic features.

The procedure followed to acquire the images was based on the study conducted by Valera-Calero et al. [26]. Participants were placed in the supine position with a pillow placed under their knees to minimize lumbar lordosis and received instructions to relax their neck muscles during the procedure (in order to avoid muscle stiffness changes due to muscle contractions [27]). Then, the transducer was placed in the supraclavicular region beside the cricoid cartilage, using enough US gel for avoiding probe compression, as this could influence SWE measurements [28]. A lateral gliding was performed until placing the carotid artery on the lateral extreme of the image. Afterwards, the transducer was glided in the cranial and caudal directions until locating the C6 transverse process in a short-axis view. This image was used as a reference since is C6 is characterized by a prominent anterior tubercle and a smaller posterior tubercle [29]. After locating C6, the probe was caudally glided until locating the transverse process of C7, which is characterized by a prominent posterior tubercle [29], to freeze the image. This scanning location showed to be highly reliable to locate the AS muscle and analyze its shape, size, and mean echo-intensity. Reliability estimates were good to excellent for test–retest measurements (either for experienced and novel examiners) and good for inter-examiner repeatability [26]. 

**Figure 1:**
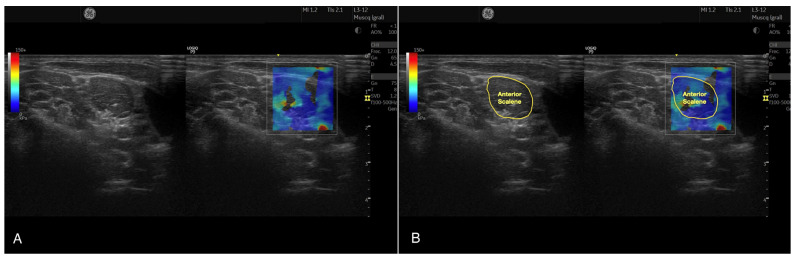
Raw B-mode Ultrasound and Shear Wave Elastography image acquired at C7 level for assessing the anterior scalene muscle (**A**) and muscle contouring for acquiring the stiffness metrics (**B**).

For acquiring the Young’s modulus and the shear wave velocity, the width and height of the region of interest was adjusted to completely cover the AS muscle. Finally, the AS muscle was carefully contoured, avoiding the inclusion of bone, nerve roots, or surrounding fascia, as illustrated in Figure 1.

### 2.5. Statistical Analysis

All statistical analyses were run using the Statistical Package for the Social Sciences (SPSS v.27, Armonk, NY, USA) for Mac, setting a two-tailed significance level of *p* < 0.05. 

First, normality of the data distribution (*p* > 0.05) was verified using histograms and Shapiro–Wilk tests. Next, the sociodemographic, body composition, and US characteristics of the overall sample were examined using descriptive statistics (mean and standard deviation for continuous, data and frequency and percentage for categorical variables). Additionally, all features were reported independently for both sexes, SWE characteristics were reported by gender and side. The Student’s *t*-test was used to analyze between-group differences in the mean difference with a 95% confidence interval, considering the differences statistically significant if *p* < 0.05.

Subsequently, test–retest reliability analyses were conducted by calculating (1) the mean average and standard deviation of both attempts for each SWE metric, (2) the absolute error between both trials (Young’s modulus error and shear wave speed error), (3) the intraclass correlation coefficients (ICC_3,1_, setting a 2-way mixed model consistency type), (4) the standard error of measurement (SEM, calculated as the standard deviation of the mean average multiplied by the square root of 1 minus ICC), and (5) the minimal detectable changes (MDC, calculated as 1.96 times the square root of 2 times SEM) [30].

Finally, a correlation matrix was constructed using Pearson’s correlation coefficients for two purposes: (1) for assessing the direction (positive values indicate proportionally directed association, and negative values proportionally undirected association) and strength (absolute r values ranging between 0 and 0.3 were considered as poor, between 0.3 and 0.6 as fair, 0.6 to 0.8 as moderate, and 0.8 to 1.0 as strong) paired associations [29], (2) identifying potential multicollinearity and shared variance among the variables (if r > 0.80) [31].

## 3. Results

Out of the 38 individuals who expressed interest in participating in the study, 2 were excluded due to a history of traumatic injury (whiplash-associated disorders) within the previous year, and 2 were lost for the second measurement. As a result, 34 asymptomatic volunteers were included in the data collection, analyzing both left and right sides of all participants. This resulted in a total of 68 AS muscles being examined, a total of 136 US images.

Table 1 summarizes the sociodemographic, body composition (both compared by gender), and AS stiffness characteristics (compared by gender and side) of the sample. The comparison between genders showed similar age, weight, and body mass index (*p* > 0.05), but men were significantly taller than women (*p* < 0.001). Despite these similarities, body composition features differed significantly between sexes. Males had less body fat mass (*p* < 0.01) and greater lean mass and water volume (both, *p* < 0.001) than women. Regarding the SWE metrics, both genders demonstrated similar AS stiffness (*p* > 0.05). No significant side-to-side asymmetries were found for Young’s modulus nor shear wave velocity (both, *p* > 0.05).

Test–retest reliability estimates for assessing AS muscle stiffness are summarized in Table 2. These results showed good-to-excellent intra-examiner reproducibility for assessing Young’s modulus (ICC = 0.857–0.946) and good-to-excellent for assessing the shear wave speed (ICC = 0.874–0.952). In addition, the SEM, MDC, and CV can be found in Table 2. 

Finally, the Pearson’s correlation matrix assessing the association among demographic characteristics, body composition features, muscle stiffness, and SWE errors is presented in Table 3. The main findings were the lack of association between age with body composition, muscle stiffness, and SWE errors (all, *p* > 0.05) and multiple associations among demographic characteristics with body composition (identifying multicollinearity and shared variance in most of the variables). Thus, no significant associations were found between muscle stiffness and SWE errors with height, weight, body mass index, water volume, lean mass, or fat mass (*p* > 0.05). In contrast, significant associations were observed between Young’s modulus errors and AS stiffness (smaller errors in stiffer muscles assessed either with Young’s modulus or shear wave speed), whereas no associations were found between shear wave speed errors and stiffness values (Young’s modulus and shear wave speed, *p* > 0.05).

## 4. Discussion

Up to the authors’ knowledge, this is the first study investigating the association between demographic and body composition features with SWE errors. However, this is not the first study analyzing the test–retest reliability of SWE for quantifying the AS stiffness [32] or aiming to analyze the association between US errors with anthropometric, demographic, and body composition characteristics [33,34]. 

From a total of two published studies found assessing the AS stiffness properties, only one focused on the test–retest reliability [32], whereas the other study provided normative values in asymptomatic subjects [35]. In the first study by Bedewi et al. [32], a preliminary assessment of test–retest reliability was conducted on a sample of 15 asymptomatic subjects to calculate the Young’s modulus at this location. The authors acknowledge that their study included a small sample size, with no minimum sample size calculation. Therefore, their results should be interpreted cautiously, as there is a considerable risk of bias associated with type II errors. 

In addition, the image acquisition procedure differed significantly from the one presented in this study. The description provided by Bedewi et al. [32] was limited to positioning the patients to the supine position and placing the probe beside the thyroid lobe. As the average length of this gland is estimated to be approximately 4.2 cm [36], the likelihood of consistently identifying the same placement point is relatively low. Despite these limitations, the authors still reported a good test–retest reliability (ICC = 0.80), and overcoming these limitations may be the reason behind the improvement in the test–retest reliability estimates obtained in this study (ICC > 0.91), as we provided a more specific and reproducible location, patient positioning, and included a larger sample size. No further comparisons between studies were possible, as Bedewi et al. [32] did not provide any score differences between the test and retest trials or any other data (i.e., SEM, MDC or CV).

The study conducted by Kuo et al. [35] provided reference values for shear wave velocity scores for orientating the normative AS muscle stiffness based on a cohort of 20 asymptomatic subjects. The study protocol described the probe placement at the lower fourth of the anterolateral aspect of the neck. However, it is important to note that this protocol may entail a considerable risk of low reproducibility due to lack of details. Yet, this hypothesis cannot be confirmed, as the authors did not provide any reliability data. 

Research analyzing morphological characteristics of the AS muscle is also scarce [26]. The procedure followed in this study for locating the AS muscle was based on the only study reporting a detailed procedure overcoming the limitations discussed before. This study found that a single measurement following this procedure was similarly reliable as calculating a mean average of two trials, both for single experienced or novel examiners performing test–retest measurements and inter-examiner agreements [26]. The main limitation of that study was the exclusive assessment of gray-scale metrics (i.e., cross-sectional area, muscle perimeter, circularity, aspect ratio, roundness, solidity, and mean echo-intensity), including no information about muscle stiffness. Although test–retest reliability estimates were better for measuring cross-sectional area (ICC = 0.954), muscle perimeter (ICC = 0.940), and mean echo-intensity (ICC = 0.969), this study found that measuring the AS muscle elasticity (ICC > 0.912) was more reliable than measuring shape descriptors such as circularity (ICC = 0.816), aspect ratio (ICC = 0.780), roundness (ICC = 0.823), and solidity (ICC = 0.766). Further research is needed to analyze the correlation between all these metrics with clinical severity indicators such as pain intensity, pain duration, neuropathic pain symptoms, disability associated with pain or pain extent in populations with neck pain with mobility deficits, movement coordination impairments, headache, or radiating pain [37]. 

While the primary aim of this study was to assess test–retest reliability and the association between SWE errors and specific features, the sample size calculation may not provide sufficient statistical power to consider the obtained scores as normative values. However, a discussion about the AS stiffness scores obtained among the studies may be of interest. This comparison is feasible despite the demographic differences among the studies, as our results, in agreement with Bedewi et al. [32] and Kuo et al. [35], showed that age, height, weight, and BMI were not significantly correlated with the AS stiffness. Mean Young’s modulus scores and their dispersions indicated that this metric was relatively consistent between Bedewi et al. [32] and our study (18.83 ± 5.32 kPa and 16.78 ± 8.99 kPa for the right side and 21.71 ± 4.8 kPa and 14.50 ± 7.57 kPa for the left side, respectively). However, there was a substantial difference in shear wave speed between the values provided by Kuo et al. [35] and our results (mean of 1.12 ± 0.17 m/s and 2.21 ± 0.56 m/s, respectively), which was likely to be explained by the methodological differences. 

Finally, it is important to highlight significant differences regarding the associations between the B-mode US and SWE errors with demographic, body composition, and anthropometric features. Varol et al. found age to be one of the most important contributors to B-mode US measurement errors for assessing cross-sectional area, circularity, aspect ratio, roundness, and mean echo intensity (defined as the average brightness intensity of pixels, measured on a 256 grayscale, over a given area) characteristics of the lumbar multifidus [33] and the mean echo-intensity of the cervical multifidus [34]. Additionally, water volume was reported to be another important contributor to B-mode measurement errors, as it was correlated with lumbar multifidus cross-sectional area errors [33] and cervical multifidus cross sectional areas, perimeters, and roundness measurement errors [34]. In parallel to our SWE results, BMI did not influence any B-mode metric measurement error in these studies [33,34]. Surprisingly, we found that these parameters were not associated with the stiffness estimation error in our study. In contrast, Young’s modulus errors (but not shear wave speed) seemed to be associated with the stiffness magnitude (i.e., stiffer muscles were more likely to exhibit greater test–retest disagreement, whereas softer muscles were prone to lead in smaller test–retest differences), as the results obtained showed greater errors in muscles with greater elasticity.

### Limitations

It is important to acknowledge several limitations of this study. One such limitation is our restriction of the sample to asymptomatic individuals. Therefore, we cannot be certain whether the reliability estimates obtained would apply to patients experiencing neck pain or thoracic outlet syndrome symptoms, especially considering that certain clinical populations have demonstrated histological changes that may impede the visualization of muscle boundaries [38,39,40]. Another limitation is that the standard deviations observed in this study are limited, and the results cannot be generalized, despite targeting a wide range of body compositions and sociodemographic factors. Therefore, future studies should include larger sample sizes with wider ranges to verify the consistency of the results reported in this study. Furthermore, a single examiner was included, as a recent study [26] stated that this procedure is acceptably reproducible among both novice and experienced examiners. However, future studies may explore whether these sociodemographic and body composition features are associated with inter-examiner disagreements.

## 5. Conclusions

This study found that a detailed procedure considering the participants’ positioning and transducer placement for locating and measuring the AS muscle stiffness demonstrated excellent reliability in asymptomatic subjects. Intra-examiner reliability was good to excellent for assessing both the Young’s modulus and shear wave speed. In addition, sociodemographic characteristics and body composition features showed no association with SWE test–retest errors. In contrast, baseline stiffness showed a significant association with SWE errors. Further research including clinical populations is needed to confirm the reliability estimates obtained in this study, analyze the discriminative capacity of SWE to detect clinical populations, and investigate the association between the AS muscle stiffness with clinical severity.

## Figures and Tables

**Table 1:** Participants’ sociodemographic and US characteristics.

Variables	Total Sample (n = 34)	Gender			Side		
		Male (n = 24)	Female (n = 10)	*Difference*	Right (n = 34)	Left (n = 34)	*Difference*
*Sociodemographic Characteristics*							
Age (y)	21.23 ± 4.75	21.91 ± 5.43	19.60 ± 1.77	2.31 (−1.29; 5.92) *p* = 0.200	-	-	-
Height (m)	1.72 ± 0.08	1.76 ± 0.06	1.65 ± 0.05	0.11 (0.07; 0.16) *p* < 0.001	-	-	-
Weight (kg)	71.95 ± 14.05	74.45 ± 13.47	65.97 ± 14.26	8.48 (−2.02; 18.99) *p* = 0.110	-	-	-
Body Mass Index (kg/m^2^)	24.10 ± 3.95	24.02 ± 3.79	24.29 ± 4.53	−0.27 (−3.34; 2.81) *p* = 0.799	-	-	-
*Body Composition Characteristics*							
Fat Mass (Kg)	16.90 ± 9.38	14.25 ± 8.24	23.25 ± 9.25	8.99 (2.44;15.54) *p* = 0.009	-	-	-
Lean Mass (Kg)	30.93 ± 6.77	34.12 ± 5.09	23.29 ± 3.17	10.83 (7.27; 14.39) *p* < 0.001	-	-	-
Water Volume (kg)	40.23 ± 8.11	44.00 ± 6.16	31.20 ± 3.97	12.80 (8.48; 17.11) *p* < 0.001	-	-	-
*Anterior Scalene Muscle Ultrasound Characteristics* ^a^							
Young’s Modulus (kPa)	15.69 ± 8.36	15.12 ± 7.76	17.42 ± 9.83	2.29 (−2.23;6.83) *p* = 0.315	16.78 ± 8.99	14. 50 ± 7.57	2.28 (−1.79; 6.36) *p* = 0.267
Shear Wave Speed (m/s)	2.21 ± 0.56	2.18 ± 0.53	2.33 ± 0.64	0.15 (−0.14;0.46) *p* = 0.312	2.29 ± 0.61	2.13 ± 0.49	0.13 (−0.11; 0.43) *p* = 0.250

^a^ Reported values are obtained by calculating the mean average of both trials.

**Table 2:** Test–retest reliability for assessing the anterior scalene muscle stiffness.

Variables	Young’s Modulus (kPa)	Shear Wave Speed (m/s)
Mean	15.69 ± 8.36	2.21 ± 0.56
Test	15.96 ± 8.91	15.42 ± 8.52
Re-Test	2.23 ± 058	2.20 ± 0.58
Absolute Difference	3.30 ± 3.71	0.21 ± 0.22
ICC_3,2_ (95% CI)	0.912 (0.857–0.946)	0.923 (0.874–0.952)
SEM	2.47	0.15
MDC_95_	3.50	0.21
CV (%)	21.0	9.5

Standard Error of Measurement (SEM) and Minimal Detectable Changes (MDC95) are expressed in the units described for each metric. CV: Coefficient of Variation; ICC: Intraclass Correlation Coefficient.

**Table 3:** Pearson’s correlation matrix.

Variables	1	2	3	4	5	6	7	8	9	10
1. Age										
2. Height	0.174									
3. Weight	0.142	0.536 **								
4. Body Mass Index	0.075	0.039	0.854 **							
5. Water Volume	0.172	0.895 **	0.744 **	0.347 **						
6. Lean Mass	0.171	0.890 **	0.739 **	0.345 **	1.000 **					
7. Fat Mass	0.014	−0.259 *	0.614 **	0.866 **	−0.071	−0.078				
8. Young’s Modulus	−0.085	−0.125	−0.102	−0.020	−0.126	−0.125	−0.004			
9. Shear Wave Speed	−0.077	−0.158	−0.117	−0.014	−0.149	−0.147	0.001	0.987 **		
10. Young’s Modulus Error	0.050	−0.157	0.052	0.197	−0.034	−0.030	0.119	0.363 **	0.390 **	
11. Shear Wave Speed Error	0.038	−0.155	0.085	0.237	−0.031	−0.026	0.164	0.198	0.212	0.927 **

Values represent Pearson’s correlation coefficients (r). * *p* < 0.05; ** *p* < 0.01.

## Data Availability

All data derived from the study are available upon reasonable request to the corresponding author.

## References

[1] Miller T., Ying M., Sau Lan Tsang C., Huang M., Pang M.Y.C. (2021). Reliability and Validity of Ultrasound Elastography for Evaluating Muscle Stiffness in Neurological Populations: A Systematic Review and Meta-Analysis. Phys. Ther..

[2] Martínez-Rodríguez R., Galán-Del-Río F., Cantalapiedra J.A., Flórez-García M.T., Martínez-Martín J., Álvaro-Meca A., Koppenhaver S.L., Fernández-de-Las-Peñas C. (2021). Reliability and discriminative validity of real-time ultrasound elastography in the assessment of tissue stiffness after calf muscle injury. J. Bodyw. Mov. Ther..

[3] Gharaibeh B., Chun-Lansinger Y., Hagen T., Ingham S.J.M., Wright V., Fu F., Huard J. (2012). Biological approaches to improve skeletal muscle healing after injury and disease. Birth Defects Res. Part C Embryo Today.

[4] Valera-Calero J.A., Sánchez-Jorge S., Buffet-García J., Varol U., Gallego-Sendarrubias G.M., Álvarez-González J. (2021). Is Shear-Wave Elastography a Clinical Severity Indicator of Myofascial Pain Syndrome? An Observational Study. J. Clin. Med..

[5] Janczyk E.M., Champigny N., Michel E., Raffaelli C., Annweiler C., Zory R., Guérin O., Sacco G. (2021). Sonoelastography to Assess Muscular Stiffness among Older Adults and its Use for the Diagnosis of Sarcopenia: A Systematic Review. Ultraschall Med..

[6] Yoshida K., Itoigawa Y., Maruyama Y., Kaneko K. (2019). Healing Process of Gastrocnemius Muscle Injury on Ultrasonography Using B-Mode Imaging, Power Doppler Imaging, and Shear Wave Elastography. J. Ultrasound Med..

[7] Snoj Ž., Wu C.H., Taljanovic M.S., Dumić-Čule I., Drakonaki E.E., Klauser A.S. (2020). Ultrasound Elastography in Musculoskeletal Radiology: Past, Present, and Future. Semin. Musculoskelet. Radiol..

[8] Ozturk A., Grajo J.R., Dhyani M., Anthony B.W., Samir A.E. (2018). Principles of ultrasound elastography. Abdom. Radiol..

[9] Taljanovic M.S., Gimber L.H., Becker G.W., Latt L.D., Klauser A.S., Melville D.M., Gao L., Witte R.S. (2017). Shear-Wave Elastography: Basic Physics and Musculoskeletal Applications. Radiographics.

[10] Sigrist R.M.S., Liau J., Kaffas A.E., Chammas M.C., Willmann J.K. (2017). Ultrasound Elastography: Review of Techniques and Clinical Applications. Theranostics.

[11] Valera-Calero J.A., Fernández-de-las-Peñas C., Fernández-Rodríguez T., Arias-Buría J.L., Varol U., Gallego-Sendarrubias G.M. (2021). Influence of Examiners’ Experience and Region of Interest Location on Semiquantitative Elastography Validity and Reliability. Appl. Sci..

[12] Koo T.K., Guo J.Y., Cohen J.H., Parker K.J. (2014). Quantifying the passive stretching response of human tibialis anterior muscle using shear wave elastography. Clin. Biomech..

[13] Koppenhaver S., Kniss J., Lilley D., Oates M., Fernández-de-Las-Peñas C., Maher R., Croy T., Shinohara M. (2018). Reliability of ultrasound shear-wave elastography in assessing low back musculature elasticity in asymptomatic individuals. J. Electromyogr. Kinesiol..

[14] Rosskopf A.B., Ehrmann C., Buck F.M., Gerber C., Flück M., Pfirrmann C.W. (2016). Quantitative Shear-Wave US Elastography of the Supraspinatus Muscle: Reliability of the Method and Relation to Tendon Integrity and Muscle Quality. Radiology.

[15] Deng W., Lin M., Yu S., Liang H., Zhang Z., Liu C. (2022). Quantifying Region-Specific Elastic Properties of Distal Femoral Articular Cartilage: A Shear-Wave Elastography Study. Appl. Bionics Biomech..

[16] DeJong H., Abbott S., Zelesco M., Spilsbury K., Martin L., Sanderson R., Ziman M., Kennedy B.F., Wood F.M. (2020). A Novel, Reliable Protocol to Objectively Assess Scar Stiffness Using Shear Wave Elastography. Ultrasound Med. Biol..

[17] Besomi M., Salomoni S.E., Hug F., Tier L., Vicenzino B., Hodges P.W. (2021). Exploration of shear wave elastography measures of the iliotibial band during different tasks in pain-free runners. Phys. Ther. Sport.

[18] Kozinc Ž., Šarabon N. (2020). Shear-wave elastography for assessment of trapezius muscle stiffness: Reliability and association with low-level muscle activity. PLoS ONE.

[19] Heneghan N.R., Smith R., Tyros I., Falla D., Rushton A. (2018). Thoracic dysfunction in whiplash associated disorders: A systematic review. PLoS ONE.

[20] Panther E.J., Reintgen C.D., Cueto R.J., Hao K.A., Chim H., King J.J. (2022). Thoracic outlet syndrome: A review. J. Shoulder Elb. Surg..

[21] Kottner J., Audigé L., Brorson S., Donner A., Gajewski B.J., Hróbjartsson A., Roberts C., Shoukri M., Streine D.L. (2011). Guidelines for Reporting Reliability and Agreement Studies (GRRAS) were proposed. J. Clin. Epidemiol..

[22] Faggion C.M. (2020). EQUATOR reporting guidelines should also be used by clinicians. J. Clin. Epidemiol..

[23] Harris R.J. (1985). A Primer of Multivariate Statistics.

[24] VanVoorhis C.W., Morgan B.L. (2007). Understanding power and rules of thumb for determining sample sizes. Tutor. Quant. Methods Psychol..

[25] Larsen M.N., Krustrup P., Araújo Póvoas S.C., Castagna C. (2021). Accuracy and reliability of the InBody 270 multi-frequency body composition analyser in 10–12-year-old children. PLoS ONE.

[26] Valera-Calero J.A., Gómez-Sánchez S., Fernández-de-Las-Peñas C., Plaza-Manzano G., Sánchez-Jorge S., Navarro-Santana M.J. (2023). A Procedure for Measuring Anterior Scalene Morphology and Quality with Ultrasound Imaging: An Intra- and Inter-rater Reliability Study. Ultrasound Med. Biol..

[27] Latash M.L. (2018). Muscle coactivation: Definitions, mechanisms, and functions. J. Neurophysiol..

[28] Groth M., Fischer L., Herden U., Brinkert F., Beime J., Deindl P., Adam G., Herrmann J. (2023). Impact of probe-induced abdominal compression on two-dimensional shear wave elastography measurement of split liver transplants in children. Rofo.

[29] Joeng E.S., Jeong Y.C., Park B.J., Kang S., Yang S.N., Yoon J.S. (2016). Sonoanatomical Change of Phrenic Nerve According to Posture During Ultrasound-Guided Stellate Ganglion Block. Ann. Rehabil. Med..

[30] Koo T.K., Li M.Y. (2016). A Guideline of Selecting and Reporting Intraclass Correlation Coefficients for Reliability Research. J. Chiropr. Med..

[31] Schober P., Boer C., Schwarte L.A. (2018). Correlation Coefficients: Appropriate Use and Interpretation. Anesth. Analg..

[32] Bedewi M.A., Alhariqi B.A., Aldossary N.M., Gaballah A.H., Sandougah K.J. (2021). Shear wave elastography of the scalene muscles in healthy adults: A preliminary study. Medicine.

[33] Varol U., Sánchez-Jiménez E., Leloup E.A.A., Navarro-Santana M.J., Fernández-de-Las-Peñas C., Sánchez-Jorge S., Valera-Calero J.A. (2023). Correlation between Body Composition and Inter-Examiner Errors for Assessing Lumbar Multifidus Muscle Size, Shape and Quality Metrics with Ultrasound Imaging. Bioengineering.

[34] Varol U., Navarro-Santana M.J., Gómez-Sánchez S., Plaza-Manzano G., Sánchez-Jiménez E., Valera-Calero J.A. (2023). Inter-Examiner Disagreement for Assessing Cervical Multifidus Ultrasound Metrics Is Associated with Body Composition Features. Sensors.

[35] Kuo W.H., Jian D.W., Wang T.G., Wang Y.C. (2013). Neck muscle stiffness quantified by sonoelastography is correlated with body mass index and chronic neck pain symptoms. Ultrasound Med. Biol..

[36] Joshi S.D., Joshi S.S., Daimi S.R., Athavale S.A. (2010). The thyroid gland and its variations: A cadaveric study. Folia Morphol..

[37] Blanpied P.R., Gross A.R., Elliott J.M., Devaney L.L., Clewley C.D., Walton D.M., Sparks C., Robertson E.K. (2017). Neck Pain: Revision 2017. J. Orthop. Sports Phys. Ther..

[38] Valera-Calero J.A., Al-Buqain-Ortega A., Arias-Buría J.L., Fernández-de-Las-Peñas C., Varol U., Ortega-Santiago R. (2021). Echo-intensity, fatty infiltration, and morphology ultrasound imaging assessment in healthy and whiplash associated disorders populations: An observational study. Eur. Spine J..

[39] Valera-Calero J.A., Úbeda-D’Ocasar E., Caballero-Corella M., Fernández-de-Las-Peñas C., Sendarrubias G.M.G., Arias-Buría J.L. (2022). Cervical Multifidus Morphology and Quality Are Not Associated with Clinical Variables in Women with Fibromyalgia: An Observational Study. Pain Med..

[40] Valera-Calero J.A., Navarro-Santana M.J., Plaza-Manzano G., Fernández-de-Las-Peñas C., Ortega-Santiago R. (2022). Identifying Demographic, Clinical, Muscular and Histological Factors Associated with Ultrasound Cervical Multifidus Measurement Errors in a Chronic Neck Pain Population. Sensors.

